# Quercetin Alleviates the Immunotoxic Impact Mediated by Oxidative Stress and Inflammation Induced by Doxorubicin Exposure in Rats

**DOI:** 10.3390/antiox10121906

**Published:** 2021-11-28

**Authors:** Mayada R. Farag, Attia A. A. Moselhy, Amany El-Mleeh, Samira H. Aljuaydi, Tamer Ahmed Ismail, Alessandro Di Cerbo, Giuseppe Crescenzo, Shimaa M. Abou-Zeid

**Affiliations:** 1 Forensic Medicine and Toxicology Department, Faculty of Veterinary Medicine, Zagazig University, Zagazig 44519, Egypt; 2 Department of Anatomy and Embryology, Faculty of Veterinary Medicine, Zagazig University, Zagazig 44519, Egypt; aameselhy@vet.zu.edu.eg; 3 Department of Pharmacology, Faculty of Veterinary Medicine, Menoufia University, Shebin Elkoum 32511, Egypt; amany.ahmed1074@vet.menofia.edu.eg; 4 Department of Biochemistry and Molecular Biology, Faculty of Veterinary Medicine, Cairo University, Giza 12211, Egypt; samira_2008m@cu.edu.eg; 5 Department of Clinical Laboratory Sciences, Turabah University College, Taif University, P.O. Box 11099, Taif 21944, Saudi Arabia; t.ismail@tu.edu.sa; 6 School of Biosciences and Veterinary Medicine, University of Camerino, 62024 Matelica, Italy; 7 Department of Veterinary Medicine, University of Bari ‘Aldo Moro’, 70121 Bari, Italy; giuseppe.crescenzo@uniba.it; 8 Department of Forensic Medicine and Toxicology, Faculty of Veterinary Medicine, University of Sadat City, Sadat City 6012201, Egypt; shaimaa.abouzaid@vet.usc.edu.eg

**Keywords:** doxorubicin, quercetin, immunoglobulins, apoptosis, spleen, cytokines

## Abstract

Doxorubicin (DOX) is a chemotherapeutic agent against hematogenous and solid tumors with undesirable side effects including immunosuppression. Quercetin (QUR), a natural flavonoid abundant in fruits and vegetables, has a potent antioxidant activity. The aim of the current study was to assess the impact of QUR on DOX-induced hematological and immunological dysfunctions in a rodent model. Randomly grouped rats were treated as follows: control, QUR alone (50 mg/kg for 15 days per os), DOX alone (2.5 mg/kg I/P, three times a week, for two weeks), and co-treated rats with QUR for 15 days prior to and concomitantly with DOX (for two weeks), at the doses intended for groups two and three. DOX alone significantly disrupted the erythrogram and leukogram variables. Serum immunoglobulin (IgG, IgM, and IgE) levels and the activities of catalase (CAT) and superoxide dismutase (SOD) in spleen were declined. The DNA damage traits in spleen were elevated with an upregulation of the expression of the apoptotic markers (p53 and Caspase-3 genes) and the proinflammatory cytokines (IL-6 and TNF-α genes), while the expression of CAT gene was downregulated. These biochemical changes were accompanied by morphological changes in the spleen of DOX-treated rats. Co-treatment with QUR abated most of the DOX-mediated alterations in hematological variables, serum immunoglobulins, and spleen antioxidant status, pro-inflammatory and apoptotic responses, and histopathological alterations. In essence, these data suggest that QUR alleviated DOX-induced toxicities on the bone marrow, spleen, and antibody-producing cells. Supplementation of chemotherapy patients with QUR could circumvent the DOX-induced inflammation and immunotoxicity, and thus prevent chemotherapy failure.

## 1. Introduction

Doxorubicin (DOX) is an anthracycline antibiotic with broad-spectrum and potent anti-neoplastic activity. It is used either alone or combined with other chemotherapeutic drugs as the frontline therapy for a wide variety of solid tumors such as breast, urogenital, gynecological, brain, and endocrine tumors, as well as lymphomas and leukemias [1]. DOX was reported to increase the production of reactive oxygen species (ROS), which may overwhelm the enzymatic antioxidants and total sulfhydryl levels present in tissues. The disruption of the oxidant-antioxidant systems in favor of the former may result in tissue injury through the formation of DNA adducts, lipid peroxidation, and protein cross-linking [2]. Unfortunately, like other anticancer drugs, DOX cannot distinguish between malignant and normal cells, so that it non-selectively induces deleterious effects in healthy tissues such as oxidative stress, inflammation, apoptosis, and mitochondrial dysfunction [1], which limit the clinical utility of the drug.

The most severe side effect of doxorubicin is cardiotoxicity, leading to life-threatening heart failure, although hepatotoxicity, nephrotoxicity, reproductive toxicity, and gastrointestinal disturbances are also common sequelae of DOX chemotherapy [3]. Aside from cardiomyopathy, the cellular elements responsible for eliciting immune responses are also affected, causing immunosuppression with increased possibility of microbial infection and wound healing delay [4,5].

Previous studies have demonstrated the toxic impacts of DOX on the hematopoietic system of rats with reduction of red blood cells (RBCs), white blood cells (WBCs), granulocytes, lymphocytes, and monocytes count [6,7]. DOX was reported to be immunosuppressive in rats, where it suppressed lymphocyte proliferation, phagocytosis activity and macrophage capacity, and CD8+ cytotoxic T cells, in addition to IL-10 downregulation [8]. In tumor bearing mice, DOX reduced the production of IL-2 and INF-γ in splenocytes and decreased lymphocyte proliferation, CD4+/CD8+ ratio, and NK cell cytotoxicity [9].

Apart from the immunosuppressive effect of DOX, the inflammatory response evoked by DOX with pro-inflammatory cytokine release has been interestingly linked to toxic effects of DOX, especially to the life-threatening cardiomyopathy, as well as liver and kidney injury [2,10,11]. Therefore, understanding DOX-induced inflammatory-immune response is essential for proper management of the toxic side effects observed during treatment especially on heart [12,13].

Natural compounds with antioxidant activity have been raising increasing interest regarding their use as possible therapeutic agents and immunostimulants [14,15,16,17,18]. It has been established that combination therapy with phytochemicals that have antioxidant and anti-inflammatory activities is beneficial in providing protection against chemotherapy-induced oxidative damage and immunomodulation [19]. Quercetin (QUR) is a plant flavonoid widely distributed in many vegetables, fruits, and seeds such as apples, cherries, grapes, onions, broccoli, peanuts, soybeans, as well as beverages of plant origin such as tea and wine [20]. It possesses antioxidant properties that may improve general health and physical/mental performance. In addition, it has antimicrobial, anti-allergic, antineoplastic, antihypertensive, and hypolipidemic properties [20,21,22].

QUR was previously reported to protect from DOX-induced cardiotoxicity and nephrotoxicity [23,24]. However, the potential of QUR to mitigate the immunotoxic effects of DOX needs to be clarified. Spleen is the largest secondary lymphoid organ which acts as a biological sieve that filters blood, and it contains high amounts of T- and B-lymphocytes, macrophages, and dendritic cells, making it crucial for regulation of innate and adaptive immune responses and elimination of pathogens [25]. Accordingly, the objective of this investigation was to assess the therapeutic potential of QUR on the DOX-induced immunotoxicity, and the underlying molecular mechanisms observed in the spleen.

## 2. Materials and Methods

### 2.1. Tested Compounds

DOX in the form of hydrochloride powder was purchased from Pharmacia Italia SPA (Benevento, Italy). QUR powder (with a purity of ≥95%) and all the other utilized chemicals of analytical grade were obtained from Sigma-Aldrich (Saint Louis, MO, USA).

### 2.2. Animals and Study Design

A total of 40 male Sprague–Dawley rats (weight: 150–200 g), obtained from the Laboratory Animal Farm at the Faculty of Veterinary Medicine, Zagazig University, were used in the present study. Rats were housed in metal cages under optimal hygienic conditions and kept at a temperature of 22–28 °C, 50% relative humidity, and exposed to 12-h light/12-h dark cycle. Food and water were provided *ad libitum* feed and water during both acclimatization (14 days) and study periods. The procedures and methodology were approved by Ethics of Animal Use in Research Committee (EAURC), at Zagazig University, Egypt (ZU- IACUC/2/F/80/2018).

Rats were randomly divided into 4 groups (10 rats/group). The first group served as control and received intraperitoneal (IP) injection with physiological saline (3 times/week, for 2 weeks). The second group (QUR) was administered QUR (50 mg/kg bw) orally via gastric tube for 15 days. The 50 mg kg^−1^ dose was chosen as this dosage was reported to prevent the side effects of DOX without affecting its antitumor activity [26] and because at a dose of 100 mg kg^−1^, no further enhancement of the protection was observed. In addition, lower (30 mg/kg) and higher doses (100 mg/kg) of QUR stimulated SIRT1 in the aorta of high-fat diet (HFD)-fed rats as well as kidney and liver of diabetic rats [27,28].

The third group (DOX) was injected with DOX intraperitoneally (IP) at a total dose of 15 mg/kg bw divided into six injections (each rat received 2.5 mg/kg bw/injection, 3 times/week, for 2 weeks), as this dose was found to induce negative effects and oxidative stress in the exposed rats and altered the functions and structure of different organs such as heart, liver and elevated left ventricular end-diastolic pressure without compromising the survival of the animals during the treatment period [29], while higher levels have been reported to affect the survivability of exposed rats [26]. The fourth group (co-treated group; QUR/DOX), received QUR orally for 15 days prior to and during the intraperitoneal administration of DOX (2 weeks). QUR was administered 1 h before injection of DOX.

### 2.3. Sampling and Analysis

At the experimental end, samples of blood were collected from the median canthus (orbital vessels) of all animals. A blood aliquot was mixed with 10% EDTA as anticoagulant for estimation of hematological parameters, while no anticoagulant was added to the remaining part of the blood sample, which was centrifuged for sera collection at 1000 rcf for 15 min. The sera were kept at −20 °C for the determination of immunological parameters.

After the blood collection, the animals were exposed to light anesthesia with diethyl-ether and then sacrificed and spleen specimens were collected, rinsed in physiological saline, and divided into 3 sets. One set was immediately immersed in liquid nitrogen and stored at −80 °C to be used for the analysis of gene expressions and DNA damage. A second set was homogenized in PBS (1:5 *w*/*v*) for 5 min and centrifuged at 4 °C for 15 min at 3000 rpm, and the supernatant was recovered and used for detection and quantification of inflammatory and antioxidant markers. The third set was fixed in neutral buffered formalin (10%) for histopathological investigations.

### 2.4. Hematological Parameters

Hemoglobin (Hb), red blood cells (RBC), white blood cells (WBC), packed cell volume (PCV), mean cell hemoglobin (MCH), and differential leukocytic count (DLC) were quantified by mean of an automatic cell counter (Hospitex Hemascreen 18-Italy). Mean corpuscular hemoglobin concentration (MCHC) and mean corpuscular volume (MCV) were calculated according to the following formulae:

MCV = Hematocrit (%) × 10/ RBCs count (millions/mm^3^ blood)

MCHC = Hemoglobin (g/100 × 100)/Hematocrit (%)

### 2.5. Assessment of Immunological Parameters in Serum

The concentrations of serum immunoglobulins; IgM, IgG and IgE were estimated by rat ELISA analytical kits (MyBioSource Co, San Diego, CA, USA, Catalog No: MBS2513365, MBS700907 and MBS705211) following the manufacturer’s instructions.

### 2.6. Antioxidants in Spleen Tissue

The catalase (CAT) and superoxide dismutase (SOD) enzymatic activities were assessed from the ablated spleen by using specific assay kits (Biodiagnostic, Giza, Egypt, Catalog No: SD2521 and CA2517), following the manufacturer’s instructions.

### 2.7. DNA Damage (Comet Assay)

DNA damage in the spleen cells was assessed according to the method of Singh et al. [30]. Briefly, cells were mounted on microscope slides and about 50 cells/slide were observed by the use of fluorescence microscope (Zeiss Axiovert Inc., Oberkochen, Germany) equipped with a CCD camera (Olympus, Japan) for imaging of cells. The DNA migration tail length, DNA%, and moment were determined for each cell. Comet Assay Project software was used to calculate the scores of tail moment from the comet images of investigated cells.

### 2.8. Transcription Levels of Antioxidant, Inflammatory Response, and Apoptosis-Related Genes by Real-Time qPCR

RNeasy Mini Kit (Qiagen, Heidelberg, Germany) was used for total RNA extraction from spleens. Then, Quantitect^®^ Reverse Transcription kit (Qiagen, Heidelberg, Germany) was utilized for the synthesis of first-strand cDNA according to the manufacturer’s protocol. The analyzed genes and the respective primers are listed in Table 1.

Then, Rotor-Gene Q instrument with a QuantiTect^®^ SYBR^®^ Green PCR kit (Qiagen, Heidelberg, Germany) was used to perform qPCR under the following conditions of amplification: 15 min at 95 °C, followed by 40 cycles of 94 °C for 15 s, 60 °C for 15 s, and 72 °C for 15 s. Then, a melt-curve analysis was performed. The relative mRNA expression patterns for all genes were then be calculated using the comparative 2^−ΔΔCt^ method [31].

### 2.9. Light Microscopy

The formalin-fixed spleen specimens were exposed to gradual ascending ethanol (70–100%) for dehydration, then cleared in xylene, and embedded in paraffin wax. The blocks were sliced into 4–5 μm sections and stained by hematoxylin and eosin (H&E) dye for histopathological investigation by the use of a light microscope (Olympus BX51 Microscope, Olympus Optical Co. Ltd, Tokyo, Japan) [32].

### 2.10. Data Analysis

Statistical comparisons were performed by one-way Analysis of Variance (ANOVA) using the SPSS 16.0 computer program (IBM, Armonk, NY, USA). Tukey’s multiple comparisons post hoc test was performed to compare mean values between treated groups and the corresponding control. The data were tested for normality by using Shapiro-Wilk W test and homogeneity of variances. A value of *p* < 0.05 was considered statistically significant.

## 3. Results

### 3.1. Effect on Hematological Variables

As displayed in Table 2, animals only treated with DOX showed a significant (*p* < 0.05) decrease in RBC count, Hb level, PCV%, MCV, and MCHC%.

The hemoglobin reduction was also significantly lower in the QUR/DOX group with respect to the control and QUR-treated group (Table 2), whereas the other variables showed only non-significant improvement. When compared with control, DOX-treated animals showed significant leukocytosis, neutrophilia, basophilia and monocytosis, as well as lymphopenia and eosinopenia.

In the co-treated group, the total leukocytic and eosinophilic counts were significantly improved in comparison to the DOX-treated group, whereas other leukogram variables showed only non-significant improvement.

### 3.2. Antioxidants in Spleen Tissue

DOX-treated rats revealed significant suppression of the activity of both SOD and CAT with respect to the control values (Table 3).

Conversely, in the co-treated group, the SOD depression was significantly mitigated if compared to the DOX group, although control value did not attain. On the other hand, CAT activity was not significantly affected in the QUR/DOX group.

### 3.3. Immunological Parameters in Serum

The DOX treated animals exhibited significant reductions in the levels of serum IgG, IgM, and IgE, in comparison with its respective control. In the QUR/DOX group, QUR was able to ameliorate these changes, compared to the DOX treated group, although, values were still significantly different from control values (Table 3).

### 3.4. DNA Damage (Comet Assay)

**Figure 1:**
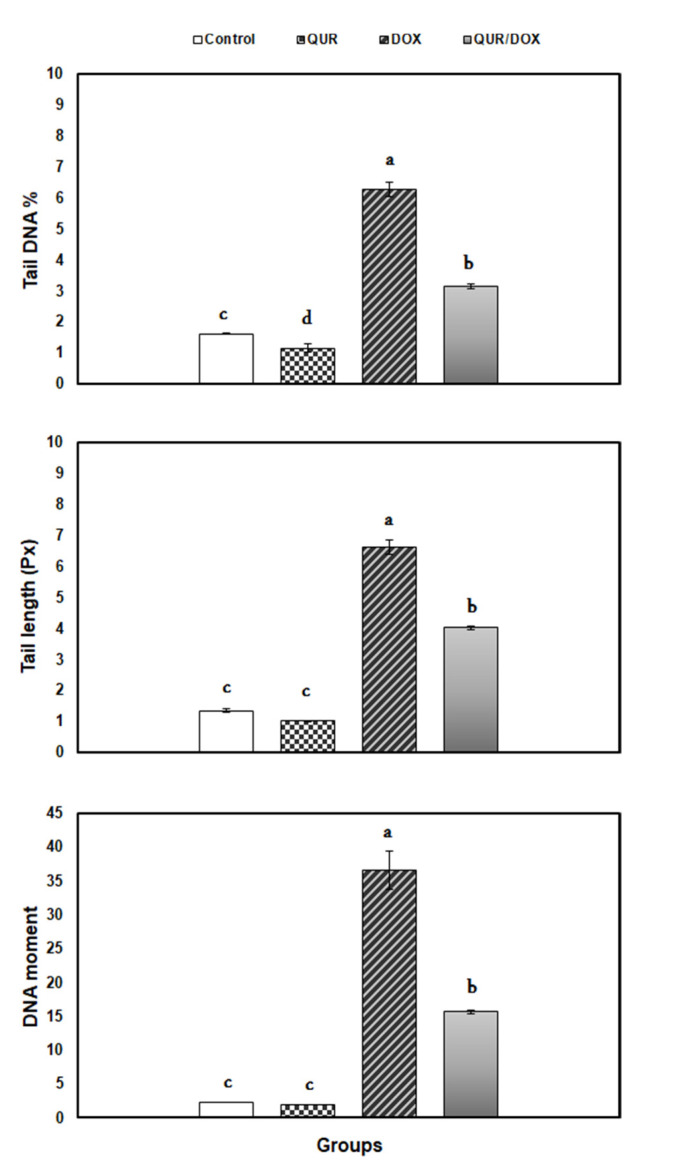
Comet variables in spleen of rats in response to QUR administration (50 mg/kg bw for 15 days, orally) and/or DOX treatment (15 mg/kg bw in six injections over 2 weeks, IP). Means with different superscripts (a,b,c,d) are significantly different (*p* < 0.05).

With respect to the control group, the comet variables: tail length, tail moment and tail DNA% exhibited significant elevation in DOX-treated group (4.91-, 3.88-, and 16.04-fold, respectively), as depicted in Figure 1.

All comet parameters of both DOX and QUR/DOX groups remained significantly different from the corresponding ones from the control group, but the animals of the co-treated group presented significant reduction of values belonging to the three indices.

### 3.5. Transcriptional Profile of Antioxidants, Inflammatory and Apoptosis-Related Genes in the Spleen Tissue

**Figure 2:**
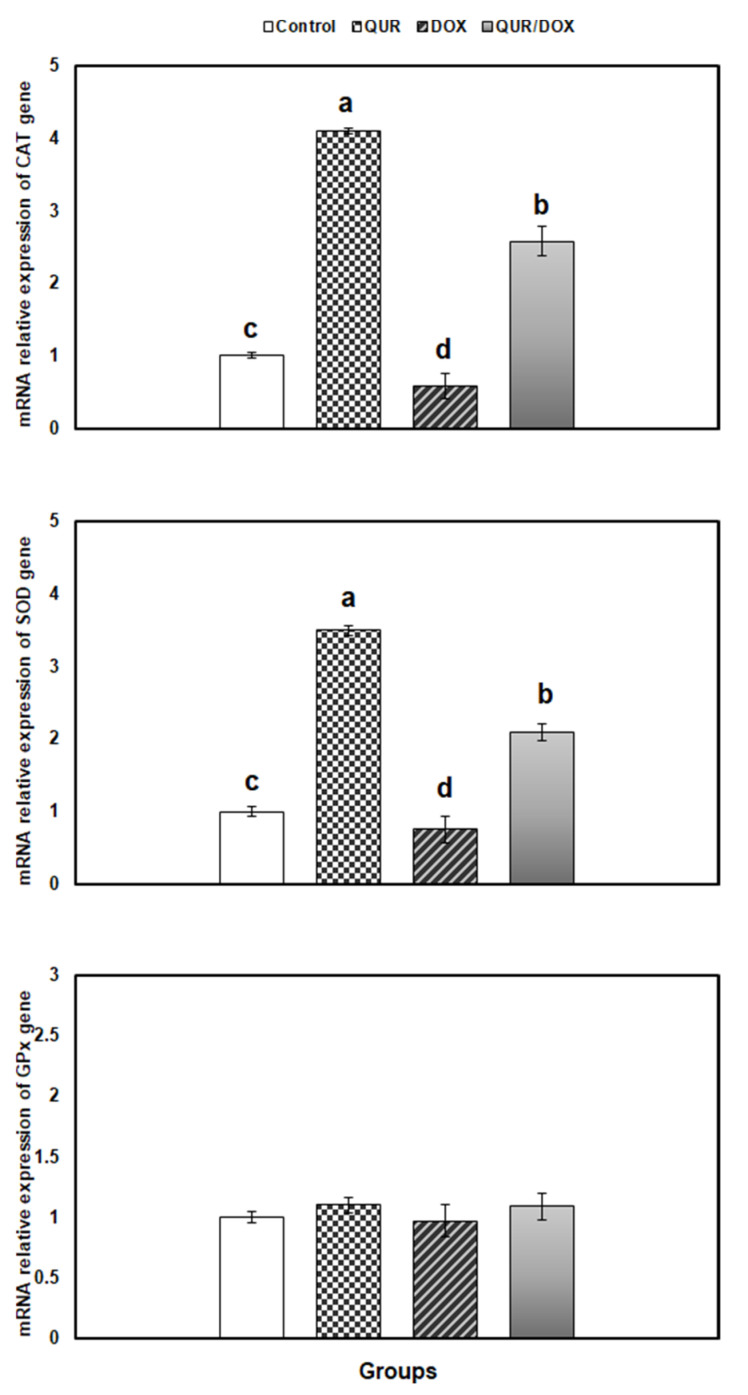
Relative expression of enzymatic antioxidant-related genes in spleen of rats in response to QUR administration (50 mg/kg bw for 15 days, orally) and/or DOX treatment (15 mg/kg bw in six injections over 2 weeks, IP). The expression pattern of genes mRNA was normalized against the internal control gene β-actin using qRT-PCR. Means with different superscripts (a,b,c,d) are significantly different (*p* < 0.05).

The impacts of QUR and/or DOX on the expression of enzymatic antioxidants in spleen are illustrated in Figure 2.

Although CAT exhibited downregulation in the DOX-treated rats (being 0.58-fold lower than control), SOD and glutathione peroxidase (GPx) showed non-significant changes, compared to control. Contrarily, coadministration of QUR with DOX reduced the CAT expression downregulation, and surprisingly showed overexpression of CAT and SOD than control genes.

**Figure 3:**
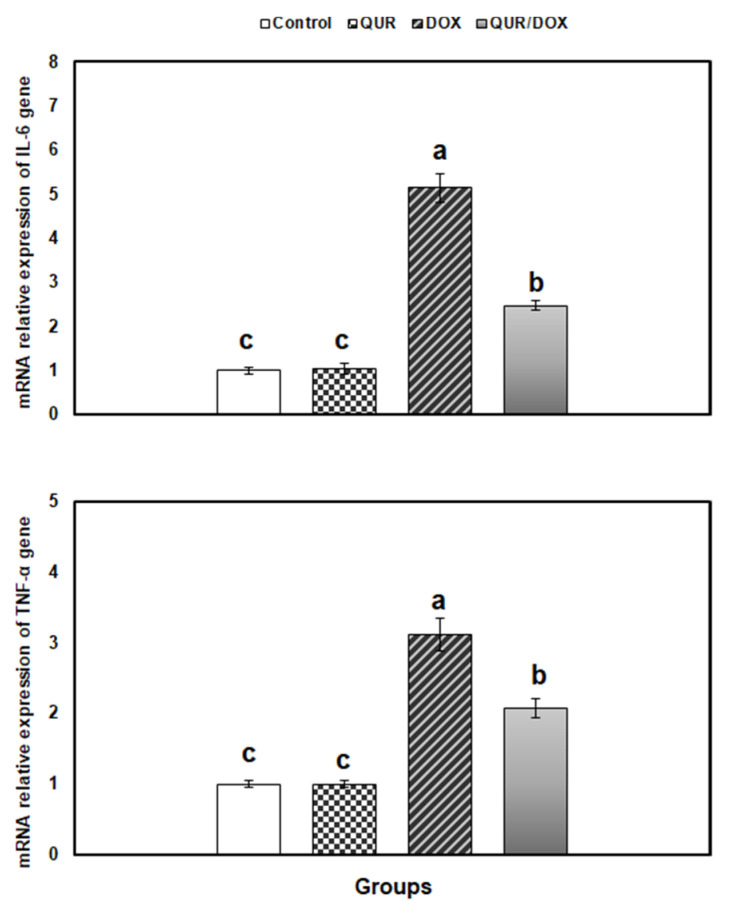
Relative expression of inflammatory response-related genes in spleen of rats in response to QUR administration (50 mg/kg bw for 15 days, orally) and/or DOX treatment (15 mg/kg bw in six injections over 2 weeks, IP). The expression pattern of genes mRNA was normalized against the internal control gene β-actin using qRT-PCR. Means with different superscripts (a,b,c) are significantly different (*p* < 0.05).

Figure 3 shows that the expressions of spleen TNF-α and IL-6 was significantly increased in the DOX-treated group by 5.14- and 3.11-fold, respectively, compared to control. This effect was significantly mitigated when QUR was administered prior to and concomitantly with DOX.

**Figure 4:**
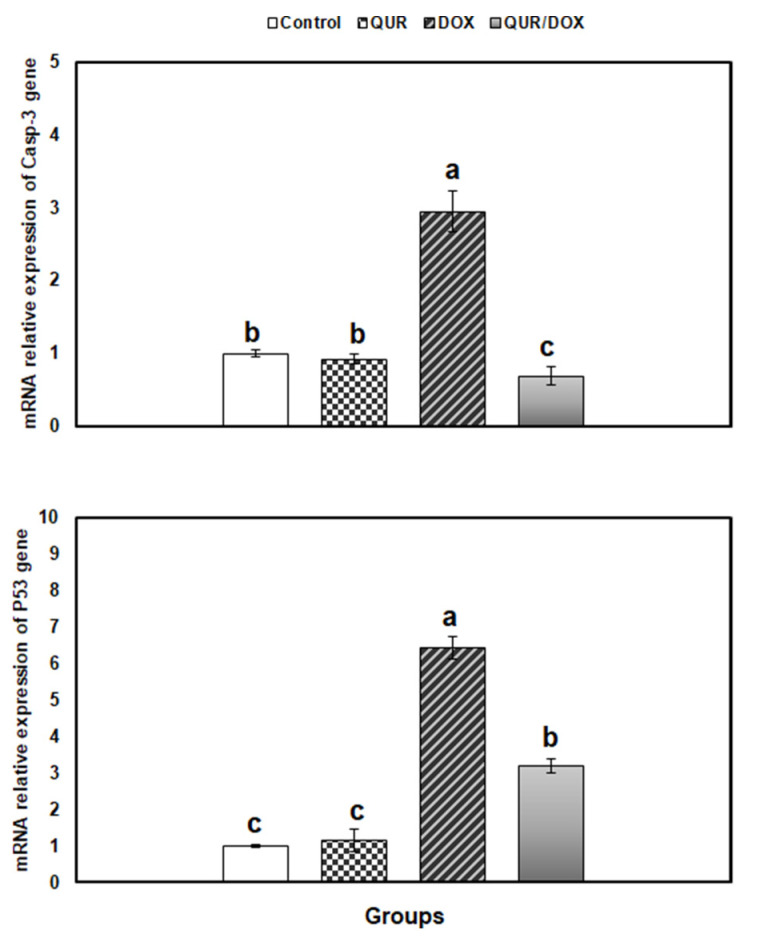
Relative expression of apoptosis-related genes in spleen of rats in response to QUR administration (50 mg/kg bw for 15 days, orally) and/or DOX treatment (15 mg/kg bw in six injections over 2 weeks, IP). The expression pattern of genes mRNA was normalized against the internal control gene β-actin using qRT-PCR. Means with different superscripts (a,b,c) are significantly different (*p* < 0.05).

The apoptosis level in spleen was assessed by measuring the relative expression of p53 and Caspase- 3 genes in the spleen. Both genes were significantly upregulated in the DOX-treated group (6.54- and 2.95-fold, respectively) than control values. Finally, in the co-treated group, a significant reduction in both gene expressions was recorded, compared to DOX group. However, only the expression level of Caspase-3 gene was restored up that of the untreated groups (Figure 4).

### 3.6. Histopathological Findings in Spleen

**Figure 5:**
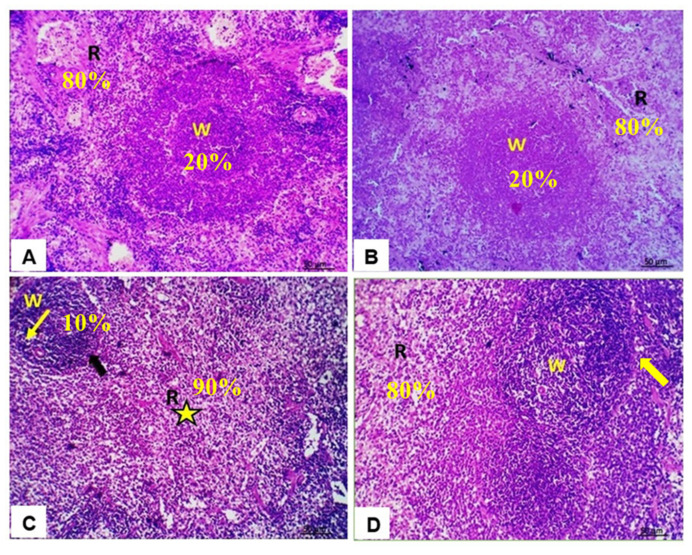
Representative histopathological photomicrographs of H&E-stained rat’s spleen. (**A**) Control showing: normal splenic parenchyma (red 80% and white pulps 20%); (**B**) QUR group showing normal splenic white (represents about 20%) and red pulps (represents about 80%); (**C**) DOX group showing shrinkage of lymphoid follicles, white pulp area with decrease in lymphocytes population (lymphocyte depletion) in the splenic periarteriolar lymphoid sheath (yellow arrow) and increased red pulp area (represents about 90%) (star); (**D**) QUR/DOX group showing somewhat normal spleen architecture with intact white pulp (W), red pulp (R) and densely cellular lymphocytes population in periarteriolar lymphoid sheath region (hyperplastic lymphoid elements in white pulps) (arrow). Scale Bar 50 μm.

To test whether QUR could ameliorate DOX-induced spleen structural alterations, H&E-stained sections were examined (Figure 5).

Control (Figure 5A) and QUR-treated (Figure 5B) rats revealed similarly intact structures with regular morphology of splenic white and red pulps. The splenic sections of the DOX-treated group showed shrinkage of lymphoid follicles, the white pulp area characterized by decrease in lymphocytes population (lymphocyte depletion) in the splenic periarteriolar lymphoid sheath and increased red pulp area (Figure 5C). Lastly, the QUR/DOX group revealed normal spleen architecture with intact white pulp, red pulp, and densely cellular lymphocytes population in the periarteriolar lymphoid sheath region (Figure 5D).

## 4. Discussion

The immunotoxic effect of DOX represents a major health concern that limits the use of DOX as a chemotherapeutic drug against various types of cancer. The results of this study provide evidence about the protective effects of QUR on DOX-induced immunotoxicity in rats, which further support the potential utility of QUR as a chemoprotective agent. This outcome is probably based on the ability of QUR to abate the DOX-induced alterations in hematological parameters, serum immunoglobulins, spleen antioxidant status, and inflammatory and apoptotic responses [24].

Our findings revealed a decline in RBC count, hemoglobin level, PCV%, MCV, and MCHC% in DOX-treated rats. These alterations could be the result of the suppression of heme synthesis and erythropoiesis or loss in the hemopoietic system [33]. Additionally, the increased rate of RBC destruction may contribute to the anemic condition, as RBCs are susceptible targets to oxidative stress [34]. Our results agree with those of Owumi et al. where rats treated with DOX showed decreased RBC, Hb, PCV, MCH, MCV, and MCHC reflecting microcytic hypochromic anemia [6]. Moreover, Bhinge et al. pointed out that DOX inhibited the bone marrow, reduced the hematopoietic stem cells and RBC count in tumor-bearing mice [35].

Blood leukocyte count and distribution reflect the general functional status of the organism and the ability to resist disturbances from the external environment. In the current study, leukocytosis, neutrophilia, basophilia, monocytosis, lymphopenia, and eosinopenia were recorded in DOX-treated animals. The observed leukocytosis and neutrophilia may be due to the DOX-induced tissue damage and enhanced inflammatory response [36]. It may also result from the disruption of normal neutrophil homeostasis causing delay of apoptosis that, in turn, will increase the life span, which might generate harmful toxic mediators [37]. The monocytosis induced by DOX may contribute to the T cell immunosuppression via inhibitory mediators that were demonstrated to inhibit the bone marrow [38].

The DOX-induced lymphopenia might be due to the inhibited production of new cells and/or destruction of mature lymphocyte populations as well as the removal of lymphocyte precursors [39]. DOX has already proved to suppress lymphocyte proliferation in a mouse-cancer model [9]. Additionally, the genotoxic and apoptotic impacts of DOX may contribute to the observed lymphopenia, as lymphocytes are particularly vulnerable to the DOX-induced DNA damage [40,41]. The lymphopenia may contribute to the suppression of humoral and/or cellular immune response, and thereby increasing vulnerability towards malignancies or infections.

Notably, QUR co-administration to rats prior to and concurrently with DOX significantly alleviated the toxic effects on hemoglobin level, and total leukocytic and eosinophilic counts. In addition, nonsignificant improvements were noticed in other erythrogram and leukogram variables. The protective effect of QUR on hematological parameters was previously reported in furan- and cadmium-intoxicated rats [42,43]. This may be attributed to the antioxidant and genoprotective effects of QUR [20]. The protective effect of QUR on various blood cells is in line with the recorded enhancement of antioxidant status as well as the alleviation of the inflammatory and apoptotic responses in the spleen of DOX-treated rats.

The immune cells are extremely sensitive to perturbation of the antioxidant status, as they play essential roles in immune response via the release of high amounts of ROS. 

Additionally, the plasma membranes of immunocytes are rich in polyunsaturated fatty acids making them more vulnerable to lipid peroxidation [44]. In this view, SOD and CAT enzymes are critical for first line defense against oxidative stress. Our data indicated inhibition of the SOD and CAT activities, and CAT expression, as well as non-significant downregulation of both SOD and GPx expression in the spleen of DOX-treated animals.

This supports the hypothesis that oxidative stress is a mechanism of DOX-induced splenotoxicity and reflects overwhelming of the antioxidant defense system due to increased generation of ROS. In accordance with our results, DOX was demonstrated to induce oxidative stress in rat spleen with reduction of CAT activity and elevation of malondialdehyde level [7]. Similarly, DOX induced oxidative injury in other tissues including the bone marrow [4], heart [9,23], and lungs [6].

When QUR was previously and concomitantly administered with DOX, it ameliorated the toxic effect on SOD activity, while that of CAT showed non-significant improvement. Interestingly, DOX upregulated the expression of both enzyme genes so that they were surprisingly higher than control values. These findings strongly suggest that QUR can relieve the DOX-induced spleen oxidative injury by enhancing the antioxidant enzyme system. In agreement with our results, QUR was reported to improve the antioxidant status and ameliorate the oxidative damage in the spleen of rotenone-treated animals and LPS-treated *Funambulus pennanti* [45,46]. Likewise, similar protective outcomes were elicited by QUR in the kidney of lead-intoxicated rats [47], and in the liver of mice treated with tert-butyl hydroperoxide [48]. The strong antioxidant effect of QUR was attributed to its hydroxyl group on the side phenyl ring, which gives it the ability to scavenge free radical, and improve enzymatic antioxidants and glutathione. The molecular mechanisms may include regulation of signaling pathways such as Nrf2/HO-1, MAPK, and TLR4/PI3K [49,50].

IgG and IgM are major immunoglobulins that are pivotal for neutralizing bacteria, viruses, toxins, along with complement activation and opsonization. IgE has established roles in hypersensitivity and allergic conditions and in the defense against parasitic worm infections [51]. Herein, serum IgG, IgM, and IgE were suppressed in DOX-treated rats, reflecting inhibition of the humoral immune response and increased susceptibility to viral and bacterial infections. This may occur due to inhibition of B-lymphocytes responsible for antibody production and/or T-helper cells that cooperate with B-cell in the antibody synthesis [36]. The recorded decline in serum immunoglobulin levels is correlated with the recorded lymphopenia and histopathological alterations in the spleen. It has been reported that high baseline IgE in patients receiving DOX and trastuzumab was accompanied by a lower risk of cardiac dysfunction [52].

Therefore, the diminished IgE level is expected to participate in the risk of cardiomyopathy in DOX-chemotherapy patients.

Consistent with our results, others have demonstrated the immunosuppressive effect of DOX such as inhibition of TCD4+ and TCD8+, and delay in rejection of a composite tissue allograft [5,53]. Similarly, Nugroho et al. showed that DOX inhibited the lymphocyte proliferation, the macrophage phagocytic activity and capacity, and CD8+ cytotoxic T cells and downregulated IL-10 [8]. The results of Jadapalli et al. provided evidence that the splenocardiac toxicity induced by DOX was marked by depletion of CD169+ macrophages that are pivotal for the start of the immune response [11].

In the QUR/DOX group, QUR was able to alleviate the decline in serum immunoglobulins, reflecting the immunostimulatory activity of on B lymphocytes.

This is likely caused, at least in part, by the antioxidant and genoprotective activities of QUR which should improve the lymphocytic count and reduce the inflammatory response and the rate of apoptosis in spleen of DOX-treated rats. Consistent with our findings, QUR improved the serum levels of IgM and IgG in furan-intoxicated rats [42].

Additionally, QUR elevated the number of IgM-producing lymphocytes [54] and serum IgG antibody titer [55] in immunized mice.

Importantly, DNA damage was reported as a main cause of inhibition of immune response. Rapid removal of injured DNA by antimutagens or exogenous DNA repair enzymes reduced the immunosuppression induced by exogenous stimuli such as UV irradiation [56]. In our study, DOX produced DNA damage in the spleen of treated rats evidenced by the alterations in the comet variables. Similarly, DOX induced DNA damage in bone marrow cells [57] and peripheral lymphocytes [41] possibly through intercalation with DNA and inhibition of topoisomerase II causing DNA double-strand breakages [1].

Co-treatment with QUR decreased the DOX-induced genotoxicity in the spleen. QUR was previously reported to relieve the oxidative DNA damage in the spleen and heart of rats treated with furan and DOX, respectively [23,42]. Moreover, Muthukumaran et al. recorded a reduction in comet parameters and micronuclei frequencies in lymphocytes from nicotine-treated rats [58]. The genoprotective effects of QUR contribute, at least partially, to the improvement of lymphopenia and spleen white pulp depletion, and consequently enhance the immune response in DOX-treated rats.

Inflammation is primarily a protective mechanism against various pathogens and is essential for repair and regeneration after tissue injury. However, uncontrolled inflammation may result in deleterious effects including immune response disruption [59].

Oxidative stress may trigger inflammatory response through activation of signaling pathways such as NF-κB. This may cause immune cell aggregation at damaged tissues with elevation in oxygen uptake, which in turn enhances the generation of ROS.

The produced inflammatory mediators recruit more inflammatory cells that, in turn, generate further ROS giving rise to a permanent oxidative and inflammatory environment [60].

As mentioned previously mentioned, IL-6 is a pleiotropic cytokine produced in response to infection and tissue damage, produced by macrophages, T and B lymphocytes, and non-immune cells. It is associated with both pro- and anti-inflammatory effects. It regulates the differentiation of monocytes into macrophages, IgG production by B cells, dendritic cell maturation, and promotion of the Th2 response via suppressing Th1 polarization [61]. 

TNF-α is a key pro-inflammatory cytokine involved in inflammatory response induction, innate immunity regulation, and control of Th1 immune response against invading pathogens [62]. Our analysis of IL-6 and TNF-α gene expression in spleen revealed overexpression of both genes in the DOX-treated rats. IL-6 may have contributed to the DOX-induced immunosuppression; since it was previously reported to participate in immunosuppression that occurred following inflammatory disorders [63]. Likewise, dysregulated TNF-α may contribute to the DOX-induced immunomodulation, since it participated in immune-inflammatory diseases such as rheumatoid arthritis and ulcerative colitis [62].

In line with our findings, DOX-treated rats displayed elevated serum levels of TNF-α, IL-6, and IL-1β, while the anti-inflammatory cytokine IL-10 was reduced [7,64].

Additionally, the kidney and lung of DOX-treated rats revealed elevation of TNF-α, IL-1β, and MPO levels [6,24]. It has been hypothesized that the DOX-induced inflammatory response may result from the activated expression of NF-κB and MAPK [65].

Interestingly, the diminished expression of both IL-6 and TNF-α genes in the combined treatment group demonstrates the anti-inflammatory activity of QUR, which could contribute to its immunostimulatory effect. QUR was reported to have an immunoregulatory activity in human dendritic cells, where it shifted the immune balance from inflammation to resolution [66]. The protective anti-inflammatory activity of QUR was previously described in a mouse nephrotoxicity model [50] and in rats and mice with chronic prostatitis and rheumatoid arthritis [67,68]. This activity of QUR was supposed to be mediated via suppression of the activation of NLRP3 inflammasome and regulating SIRT1 pathway [69], and downregulation of NF-κB [50], inq21f, and MAPK signaling pathways [67].

Apoptosis is a physiological self-regulatory mechanism of cell death to maintain homeostasis in multicellular organisms. It may be triggered by the extrinsic (death receptor-dependent) and the intrinsic (mitochondrial-dependent) pathways, both of which are associated with activation of caspases [70]. Apoptosis consequent to oxidative damage is largely due to the intrinsic pathway activation at the mitochondrial level. Initially, mitochondrial membrane permeability is disrupted by ROS through Bax activation and Bcl-2 suppression, with releasing of cytochrome-c into the cytosol, which activates caspase-9 to generate the apoptosome with subsequent activation of the caspase-3 which is implicated in full apoptosis of cell [71].

Our results indicated overexpression of splenic p53 and Caspase-3 and in the DOX-treated rats. p53 is involved in controlling apoptosis and proliferation, so that, when overexpressed, it induces apoptosis directly by Bax-activation, or by suppressing the protein Bcl-2 protein [72]. The recorded change in apoptosis markers is consistent with the observed alterations in the antioxidant status in the spleen of DOX-treated rats. 

Our results are also in agreement with those of Shaldoum et al., who found that DOX upregulated the expressions of Caspase-3, Bax, and p-53 genes, and increased Bax/Bcl-2, while downregulated Bcl-2 gene expression in both spleen and bone marrow of rats [7]. Similarly, Owumi et al. demonstrated elevated Caspase-3 activity in the lung of DOX-treated rats [6].

Co-administration of QUR attenuated the apoptotic activity of DOX in the spleen. This might explain the immunostimulatory effect of QUR due to the inhibition of lymphocyte and other immunocyte apoptosis. Such hypothesis is supported by findings of Kumar et al., who reported that QUR suppressed the increased caspase activity in the spleen and thymus, and thus alleviated the immunotoxic effect of deltamethrin in rats [73].

Also, QUR suppressed the microcystin-induced DNA fragmentation in lymphocytes in vitro [74]. Furthermore, QUR supplementation attenuated the increased activity of caspases-9 and −3, Bax, and p53 transcripts in the kidney of copper-treated mice [50]. The anti-apoptotic activity of QUR is suggested to be mediated by down-regulation of the pro-apoptotic proteins such as Bax, and up-regulation of the anti-apoptotic proteins such as Bcl-2 [50].

In the current study, the DOX-treated group presented morphological alterations in spleen sections. Previous studies showed that DOX caused splenic hypoplasia in mice [11] and reduced the volumetric density of white pulp and white pulp/red pulp ratio in rats [7].

The observed depletion in spleen white pulp could result from suppression of lymphoproliferation [75], and the DOX-induced genotoxic effect with possible cytotoxicity.

Co-treatment with QUR mitigated the spleen morphological changes induced by DOX, which could result from QUR antioxidant, anti-inflammatory, anti-apoptotic effects on splenocytes. 

## 5. Conclusions

Based on the present findings, the protective effects of QUR against DOX-induced immunotoxicity could be a combination of QUR ability to improve the antioxidant status and to inhibit the inflammatory response and proapoptotic proteins in the spleen. QUR might be administered as a dietary supplement to alleviate the unwanted immunotoxicity and inflammatory responses in patients exposed to DOX chemotherapy, which is expected to prevent chemotherapy failure.

Additional studies are needed to gain greater insight into the molecular mechanistic pathways underlying the immunoprotective effect of QUR in DOX-treated animals.

## Figures and Tables

**Table 1:** Primers sequences used for real time PCR analysis.

Gene	Sequence	5′-3′ Primer Sequence	Accession Number
Tumor necrosis factor-alpha (TNFα)	forward	GTTCTCTTCAAGGGACAAGGC	NM_012675.3
	reverse	TGGAAGACTCCTCCCAGGTA	
Interleukin-6 (IL6)	forward	CCACTGCCTTCCCTACTTCA	NM_012589.2
	reverse	ACAGTGCATCATCGCTGTTC	
Tumor suppressor P53	forward	CTGTTTCAAAAAGCAAAAAGATGAC	NM_030989.3
	reverse	TAGCAAGGAAAGTCATGAACTGCCA	
Caspase 3, apoptosis-related cysteine peptidase (Caspase 3)	forward	TTTGCGCCATGCTGAAACT	NM_012922.2
	reverse	ACGAGTGAGGATGTGCATGAATT	
Superoxide dismutase (SOD)	forward	CATTCCATCATTGGCCGTACT	BC_082800.1
	reverse	CCACCTTTGCCCAAGTCATC	
Catalase (CAT)	forward	GTACAGGCCGGCTCTCACA	NM_012520.2
	reverse	ACCCGTGCTTTACAGGTTAGCT	
Glutathione peroxidase (GPx)	forward	GCGCTGGTCTCGTCCATT	NM_030826.3
	reverse	TGGTGAAACCGCCTTTCTTT	
Beta actin (β-actin)	forward	CACCATGTACCCAGGCATTG	NM_031144.3
	reverse	CCTGCTTGCTGATCCACATC	

**Table 2:** Hematological parameters in rats in response to QUR administration (50 mg/kg bw for 15 days, orally) and/or DOX treatment (15 mg/kg bw in six injections over 2 weeks, IP).

Hematological Variables	Experimental Groups			
	Control	QUR	DOX	QUR/DOX
RBCs (10^6^/mm^3^)	8.19 ± 0.12 ^a^	8.13 ± 0.41 ^a^	5.53 ± 0.69 ^b^	6.56 ± 0.38 ^b^
Hb (g/dl)	13.77 ± 0.03 ^a^	13.62 ± 0.24 ^a^	8.46 ± 0.07 ^c^	9.48 ± 0.20 ^b^
PCV (%)	48.37 ± 2.92 ^a^	49.86 ± 0.24 ^a^	32.05 ± 2.66 ^b^	28.68 ± 2.64 ^b^
MCV (fl)	52.47 ± 1.38 ^a^	51.96 ± 0.79 ^a^	36.98 ± 2.41 ^b^	40.17 ± 0.73 ^b^
MCH (Pg)	34.25 ± 1.95 ^a^	35.29 ±1.28 ^a^	23.30 ± 1.45 ^b^	26.46 ± 1.11 ^b^
MCHC (%)	8.74 ± 0.13 ^c^	9.40 ± 0.70 ^c^	12.90 ±0.03 ^a^	11.26 ± 0.85 ^b^
WBC (10^3^/mm^3^)	4.75 ± 0.03 ^a^	4.71 ± 0.05 ^a^	3.13 ± 0.49 ^b^	2.96 ± 0.31 ^b^
Lymphocyte (10^3^/mm^3^)	4.28 ± 0.39 ^b^	4.05 ± 0.24 ^b^	8.14 ± 0.58 ^a^	7.08 ± 0.41 ^a^
Neutrophil (10^3^/mm^3^)	0.803 ± 0.00 ^a^	0.810 ± 0.00 ^a^	0.383 ± 0.04 ^b^	0.727 ± 0.05 ^a^
Eosinophil (10^3^/mm^3^)	0.03 ± 0.00 ^b^	0.04 ± 0.00 ^b^	0.05 ± 0.00 ^a^	0.05 ± 0.00 ^a^
Basophil (10^3^/mm^3^)	0.11 ± 0.013 ^b^	0.12 ± 0.013 ^b^	1.75 ± 0.09 ^a^	1.723 ± 0.06 ^a^
Monocyte (10^3^/mm^3^)	8.19 ± 0.12 ^a^	8.13 ± 0.41 ^a^	5.53 ± 0.69 ^b^	6.56 ± 0.38 ^b^

Means within the same row (in each parameter) with different superscripts (^a,b,c^) are significantly different (*p* < 0.05) (mean ± SEM). QUR: Quercetin, DOX: Doxorubicin, QUR/DOX: Prophylaxis co-treated group, bw: body weight, IP: intraperitoneally.

**Table 3:** Antioxidant and immunological indices in rats in response to QUR administration (50 mg/kg bw for 15 days, orally) and/or DOX treatment (15 mg/kg bw in six injections over 2 weeks, IP).

Variables	Experimental Groups			
	Control	QUR	DOX	QUR/DOX
SOD (U/g tissue)	12.39 ± 0.44 ^a^	13.27 ± 0.39 ^a^	8.29 ± 0.52 ^c^	9.99 ± 0.19 ^b^
CAT (U/g tissue)	2.51 ± 0.09 ^a^	2.63 ± 0.15 ^a^	1.64 ± 0.19 ^b^	1.64 ± 0.052 ^b^
IgG (mg/dl)	44.91 ± 0.84 ^a^	46.44 ± 0.42 ^a^	16.36 ± 0.11 ^c^	36.97 ± 0.08 ^b^
IgM (mg/dl)	71.73 ± 0.82 ^a^	72.92 ± 0.72 ^a^	52.21 ± 0.43 ^c^	67.94 ± 0.91 ^b^
IgE (mg/dl)	18.07 ± 0.50 ^a^	17.27 ± 0.04 ^a^	6.50 ± 0.09 ^c^	13.13 ± 0.05 ^b^

Means within the same row (in each parameter) with different superscripts (^a,b,c^) are significantly different (*p* < 0.05) (mean ± SEM). QUR: Quercetin, DOX: Doxorubicin, QUR/DOX: Prophylaxis co-treated group, bw: body weight, IP: intraperitoneally.

## Data Availability

Data is contained within the article.

## References

[1] Sritharan S., Sivalingam N. (2021). A comprehensive review on time-tested anticancer drug doxorubicin. Life Sci..

[2] Prasanna P.L., Renu K., Gopalakrishnan A.V. (2020). New molecular and biochemical insights of doxorubicin-induced hepatotoxicity. Life Sci..

[3] Tacar O., Sriamornsak P., Dass C.R. (2013). Doxorubicin: An update on anticancer molecular action, toxicity and novel drug delivery systems. J. Pharm. Pharmacol..

[4] Jagetia G.C., Hmingthazuali V.L. (2018). Protection of Doxorubicin-Induced Biochemical Injury in the Rat Bone Marrow by a Dietary Bioflavonoid Naringin. Ann. Clin. Lab. Res..

[5] Lubis M.R., Haryani R., Safriana S., Satria D. (2019). Ethanolic Extract of Herb Pugun Tanoh (Picria fel-terrae Lour.) Modulates TCD4+ and TCD8+ Cell Profile of Doxorubicin-Induced Immuno-Suppressed Rats. Open Access Maced. J. Med. Sci..

[6] Owumi S.E., Nwozo S.O., Arunsi U.O., Oyelere A.K., Odunola O.A. (2021). Co-administration of Luteolin mitigated toxicity in rats’ lungs associated with doxorubicin treatment. Toxicol. Appl. Pharmacol..

[7] Shaldoum F., El-Kott A.F., Ouda M.M.A., Abd-Ella E.M. (2021). Immunomodulatory effects of bee pollen on doxorubicin-induced bone marrow/spleen immunosuppression in rat. J. Food Biochem..

[8] Nugroho A.E., Hermawan A., Nastiti K., Suven S., Elisa P., Hadibarata T., Meiyanto E. (2012). Immunomodulatory effects of hexane insoluble fraction of Ficus septica Burm. F. in doxorubicin-treated rats. Asian Pac. J. Cancer Prev..

[9] Zhang X.Y., Li W.G., Wu Y.J., Gao M.T. (2005). Amelioration of doxorubicin-induced myocardial oxidative stress and immunosuppression by grape seed proanthocyanidins in tumour-bearing mice. J. Pharm. Pharmacol..

[10] Carvalho C., Santos R.X., Cardoso S., Correia S., Oliveira P.J., Santos M.S., Moreira P.I. (2009). Doxorubicin: The good, the bad and the ugly effect. Curr. Med. Chem..

[11] Jadapalli J.K., Wright G.W., Kain V., Sherwani M.A., Sonkar R., Yusuf N., Halade G.V. (2018). Doxorubicin triggers splenic contraction and irreversible dysregulation of COX and LOX that alters the inflammation-resolution program in the myocardium. Am. J. Physiol. Heart Circ. Physiol..

[12] Zhang S., You Z.Q., Yang L., Li L.L., Wu Y.P., Gu L.Q., Xin Y.F. (2019). Protective effect of Shenmai injection on doxorubicin-induced cardiotoxicity via regulation of inflammatory mediators. BMC Complement. Altern. Med..

[13] Guo J., Cao W., Chen C., Chen X. (2020). Peroxiredoxin 6 overexpression regulates adriamycin-induced myocardial injury, oxidative stress and immune response in rats. Ann. Transl. Med..

[14] Khalil S.R., Abdel-Motal S.M., Abd-Elsalam M., Abd El-Hameed N.E., Awad A. (2020). Restoring strategy of ethanolic extract of Moringa oleifera leaves against Tilmicosin-induced cardiac injury in rats: Targeting cell apoptosis-mediated pathways. Gene.

[15] Khalil S.R., Salem H.F.A., Metwally M.M.M., Emad R.M., Elbohi K.M., Ali S.A. (2020). Protective effect of Spirulina platensis against physiological, ultrastructural and cell proliferation damage induced by furan in kidney and liver of rat. Ecotoxicol. Environ. Saf..

[16] Abou-Zeid S.M., Ahmed A.I., Awad A., Mohammed W.A., Metwally M.M.M., Almeer R., Abdel-Daim M.M., Khalil S.R. (2021). Moringa oleifera ethanolic extract attenuates tilmicosin-induced renal damage in male rats via suppression of oxidative stress, inflammatory injury, and intermediate filament proteins mRNA expression. Biomed. Pharmacother..

[17] El Bohi K.M., Abdel-Motal S.M., Khalil S.R., Abd-Elaal M.M., Metwally M.M.M., El Hady W.M. (2021). The efficiency of pomegranate (Punica granatum) peel ethanolic extract in attenuating the vancomycin-triggered liver and kidney tissues injury in rats. Environ. Sci. Pollut. Res. Int..

[18] Farag M.R., Alagawany M., Taha H.S.A., Ismail T.A., Khalil S.R., Abou-Zeid S.M. (2021). Immune response and susceptibility of Nile tilapia fish to Aeromonas hydrophila infection following the exposure to Bifenthrin and/or supplementation with Petroselinum crispum essential oil. Ecotoxicol. Environ. Saf..

[19] Kaiserova H., Simunek T., van der Vijgh W.J., Bast A., Kvasnickova E. (2007). Flavonoids as protectors against doxorubicin cardiotoxicity: Role of iron chelation, antioxidant activity and inhibition of carbonyl reductase. Biochim. Biophys. Acta.

[20] Karimi A., Naeini F., Azar V.A., Hasanzadeh M., Ostadrahimi A., Niazkar H.R., Mobasseri M., Tutunchi H. (2021). A comprehensive systematic review of the therapeutic effects and mechanisms of action of quercetin in sepsis. Phytomedicine.

[21] D’Andrea G. (2015). Quercetin: A flavonol with multifaceted therapeutic applications?. Fitoterapia.

[22] Li Y., Yao J., Han C., Yang J., Chaudhry M.T., Wang S., Liu H., Yin Y. (2016). Quercetin, Inflammation and Immunity. Nutrients.

[23] Zakaria N., Khalil S.R., Awad A., Khairy G.M. (2018). Quercetin Reverses Altered Energy Metabolism in the Heart of Rats Receiving Adriamycin Chemotherapy. Cardiovasc. Toxicol..

[24] Khalil S.R., Mohammed A.T., Abd El-Fattah A.H., Zaglool A.W. (2018). Intermediate filament protein expression pattern and inflammatory response changes in kidneys of rats receiving doxorubicin chemotherapy and quercetin. Toxicol. Lett..

[25] Willard-Mack C.L., Elmore S.A., Hall W.C., Harleman J., Kuper C.F., Losco P., Rehg J.E., Ruhl-Fehlert C., Ward J.M., Weinstock D. (2019). Nonproliferative and Proliferative Lesions of the Rat and Mouse Hematolymphoid System. Toxicol. Pathol..

[26] Sanchez-Gonzalez P.D., Lopez-Hernandez F.J., Perez-Barriocanal F., Morales A.I., Lopez-Novoa J.M. (2011). Quercetin reduces cisplatin nephrotoxicity in rats without compromising its anti-tumour activity. Nephrol. Dial. Transplant..

[27] Iskender H., Dokumacioglu E., Sen T.M., Ince I., Kanbay Y., Saral S. (2017). The effect of hesperidin and quercetin on oxidative stress, NF-kappaB and SIRT1 levels in a STZ-induced experimental diabetes model. Biomed. Pharmacother..

[28] Zhang F., Feng J., Zhang J., Kang X., Qian D. (2020). Quercetin modulates AMPK/SIRT1/NF-kappaB signaling to inhibit inflammatory/oxidative stress responses in diabetic high fat diet-induced atherosclerosis in the rat carotid artery. Exp. Ther. Med..

[29] Siveski-Iliskovic N., Kaul N., Singal P.K. (1994). Probucol promotes endogenous antioxidants and provides protection against adriamycin-induced cardiomyopathy in rats. Circulation.

[30] Singh N.P., McCoy M.T., Tice R.R., Schneider E.L. (1988). A simple technique for quantitation of low levels of DNA damage in individual cells. Exp. Cell Res..

[31] Livak K.J., Schmittgen T.D. (2001). Analysis of relative gene expression data using real-time quantitative PCR and the 2(-Delta Delta C(T)) Method. Methods.

[32] Suvarna K.S., Layton C., Bancroft J.D. (2018). Bancroft’s Theory and Practice of Histological Techniques E-Book.

[33] Chinde S., Grover P. (2017). Toxicological assessment of nano and micron-sized tungsten oxide after 28days repeated oral administration to Wistar rats. Mutat. Res. Genet. Toxicol. Environ. Mutagen..

[34] Mansour S.A., Mossa A.-T.H. (2009). Lipid peroxidation and oxidative stress in rat erythrocytes induced by chlorpyrifos and the protective effect of zinc. Pestic. Biochem. Physiol..

[35] Bhinge K.N., Gupta V., Hosain S.B., Satyanarayanajois S.D., Meyer S.A., Blaylock B., Zhang Q.J., Liu Y.Y. (2012). The opposite effects of doxorubicin on bone marrow stem cells versus breast cancer stem cells depend on glucosylceramide synthase. Int. J. Biochem. Cell Biol..

[36] Aroonvilairat S., Tangjarukij C., Sornprachum T., Chaisuriya P., Siwadune T., Ratanabanangkoon K. (2018). Effects of topical exposure to a mixture of chlorpyrifos, cypermethrin and captan on the hematological and immunological systems in male Wistar rats. Environ. Toxicol. Pharmacol..

[37] Nemmar A., Melghit K., Ali B.H. (2008). The acute proinflammatory and prothrombotic effects of pulmonary exposure to rutile TiO_2_ nanorods in rats. Exp. Biol. Med..

[38] Tsang Y.-W., Chi K.-H., Hu C.-J., Tseng C.-L., Tseng F.-W., Wang Y.-S. (2007). Chemotherapy-induced immunosuppression is restored by a fermented soybean extract: A proof of concept clinical trial. Nutr. Res..

[39] Steele T.A. (2002). Chemotherapy-induced immunosuppression and reconstitution of immune function. Leuk. Res..

[40] Ferraro C., Quemeneur L., Prigent A.F., Taverne C., Revillard J.P., Bonnefoy-Berard N. (2000). Anthracyclines trigger apoptosis of both G0-G1 and cycling peripheral blood lymphocytes and induce massive deletion of mature T and B cells. Cancer Res..

[41] Katoch O., Kumar A., Adhikari J.S., Dwarakanath B.S., Agrawala P.K. (2013). Sulforaphane mitigates genotoxicity induced by radiation and anticancer drugs in human lymphocytes. Mutat. Res. Genet. Toxicol. Environ. Mutagen..

[42] Alam R.T., Zeid E.H., Imam T.S. (2017). Protective role of quercetin against hematotoxic and immunotoxic effects of furan in rats. Environ. Sci. Pollut. Res. Int..

[43] Donmez H.H., Donmez N., Kisadere I., Undag I. (2019). Protective effect of quercetin on some hematological parameters in rats exposed to cadmium. Biotech. Histochem..

[44] Ince E. (2020). The protective effect of quercetin in the alcohol-induced liver and lymphoid tissue injuries in newborns. Mol. Biol. Rep..

[45] Rastogi S., Haldar C. (2018). Comparative effect of melatonin and quercetin in counteracting LPS induced oxidative stress in bone marrow mononuclear cells and spleen of Funambulus pennanti. Food Chem. Toxicol..

[46] Akinmoladun A.C., Olaniyan O.O., Famusiwa C.D., Josiah S.S., Olaleye M.T. (2020). Ameliorative effect of quercetin, catechin, and taxifolin on rotenone-induced testicular and splenic weight gain and oxidative stress in rats. J. Basic Clin. Physiol. Pharmacol..

[47] Liu C.M., Sun Y.Z., Sun J.M., Ma J.Q., Cheng C. (2012). Protective role of quercetin against lead-induced inflammatory response in rat kidney through the ROS-mediated MAPKs and NF-κB pathway. Biochim. Biophys. Acta.

[48] Kalantari H., Foruozandeh H., Khodayar M.J., Siahpoosh A., Saki N., Kheradmand P. (2018). Antioxidant and hepatoprotective effects of Capparis spinosa L. fractions and Quercetin on tert-butyl hydroperoxide- induced acute liver damage in mice. J. Tradit. Complement. Med..

[49] Xu D., Hu M.J., Wang Y.Q., Cui Y.L. (2019). Antioxidant Activities of Quercetin and Its Complexes for Medicinal Application. Molecules.

[50] Peng X., Dai C., Zhang M., Das Gupta S. (2020). Molecular Mechanisms Underlying Protective Role of Quercetin on Copper Sulfate-Induced Nephrotoxicity in Mice. Front. Vet. Sci..

[51] Schroeder H.W., Cavacini L. (2010). Structure and function of immunoglobulins. J. Allergy Clin. Immunol..

[52] Beer L.A., Kossenkov A.V., Liu Q., Luning Prak E., Domchek S., Speicher D.W., Ky B. (2016). Baseline Immunoglobulin E Levels as a Marker of Doxorubicin- and Trastuzumab-Associated Cardiac Dysfunction. Circ. Res..

[53] Hui-Chou H.G., Olenczak J.B., Drachenberg C.B., Shea S.M., Rodriguez E.D. (2012). Short-term application of doxorubicin chemotherapy immunosuppressive side effects for composite tissue allotransplantation. Ann. Plast. Surg..

[54] Valentova K., Sima P., Rybkova Z., Krizan J., Malachova K., Kren V. (2016). (Anti)mutagenic and immunomodulatory properties of quercetin glycosides. J. Sci. Food Agric..

[55] Singh D., Tanwar H., Jayashankar B., Sharma J., Murthy S., Chanda S., Singh S.B., Ganju L. (2017). Quercetin exhibits adjuvant activity by enhancing Th2 immune response in ovalbumin immunized mice. Biomed. Pharmacother..

[56] Katiyar S.K., Vaid M., van Steeg H., Meeran S.M. (2010). Green tea polyphenols prevent UV-induced immunosuppression by rapid repair of DNA damage and enhancement of nucleotide excision repair genes. Cancer Prev. Res..

[57] Uspenskaya Y.A., Mikhutkina S.V., Taksanova E.I., Popova N.N., Olovyannikova R.Y., Salmina A.B. (2004). Induction of apoptosis in bone marrow cells is mediated via purinergic receptors. Bull. Exp. Biol. Med..

[58] Muthukumaran S., Sudheer A.R., Nalini N., Menon V.P. (2008). Effect of quercetin on nicotine-induced biochemical changes and DNA damage in rat peripheral blood lymphocytes. Redox Rep..

[59] Oyinloye B.E., Adenowo A.F., Kappo A.P. (2015). Reactive oxygen species, apoptosis, antimicrobial peptides and human inflammatory diseases. Pharmaceuticals.

[60] Reuter S., Gupta S.C., Chaturvedi M.M., Aggarwal B.B. (2010). Oxidative stress, inflammation, and cancer: How are they linked?. Free Radic. Biol. Med..

[61] Velazquez-Salinas L., Verdugo-Rodriguez A., Rodriguez L.L., Borca M.V. (2019). The Role of Interleukin 6 during Viral Infections. Front. Microbiol..

[62] Silva L.C., Ortigosa L.C., Benard G. (2010). Anti-TNF-alpha agents in the treatment of immune-mediated inflammatory diseases: Mechanisms of action and pitfalls. Immunotherapy.

[63] Kanterman J., Sade-Feldman M., Baniyash M. (2012). New insights into chronic inflammation-induced immunosuppression. Semin. Cancer Biol..

[64] Tien C.C., Peng Y.C., Yang F.L., Subeq Y.M., Lee R.P. (2016). Slow infusion rate of doxorubicin induces higher pro-inflammatory cytokine production. Regul. Toxicol. Pharmacol..

[65] Wallace K.B., Sardao V.A., Oliveira P.J. (2020). Mitochondrial Determinants of Doxorubicin-Induced Cardiomyopathy. Circ. Res..

[66] Michalski J., Deinzer A., Stich L., Zinser E., Steinkasserer A., Knippertz I. (2020). Quercetin induces an immunoregulatory phenotype in maturing human dendritic cells. Immunobiology.

[67] Meng L.Q., Yang F.Y., Wang M.S., Shi B.K., Chen D.X., Chen D., Zhou Q., He Q.B., Ma L.X., Cheng W.L. (2018). Quercetin protects against chronic prostatitis in rat model through NF-κB and MAPK signaling pathways. Prostate.

[68] Yuan K., Zhu Q., Lu Q., Jiang H., Zhu M., Li X., Huang G., Xu A. (2020). Quercetin alleviates rheumatoid arthritis by inhibiting neutrophil inflammatory activities. J. Nutr. Biochem..

[69] Zhang Y., Qu X., Gao H., Zhai J., Tao L., Sun J., Song Y., Zhang J. (2020). Quercetin attenuates NLRP3 inflammasome activation and apoptosis to protect INH-induced liver injury via regulating SIRT1 pathway. Int. Immunopharmacol..

[70] Kroemer G., Galluzzi L., Brenner C. (2007). Mitochondrial membrane permeabilization in cell death. Physiol. Rev..

[71] Elmore S. (2007). Apoptosis: A review of programmed cell death. Toxicol. Pathol..

[72] Heo K.S., Lee H., Nigro P., Thomas T., Le N.T., Chang E., McClain C., Reinhart-King C.A., King M.R., Berk B.C. (2011). PKCζ mediates disturbed flow-induced endothelial apoptosis via p53 SUMOylation. J. Cell Biol..

[73] Kumar A., Gupta M., Sharma R., Sharma N. (2020). Deltamethrin-Induced Immunotoxicity and its Protection by Quercetin: An Experimental Study. Endocr. Metab. Immune. Disord. Drug Targets.

[74] Zhang H., Wu Y., Fang W., Wang D. (2014). Regulatory effect of quercetin on hazardous microcystin-LR-induced apoptosis of Carassius auratus lymphocytes in vitro. Fish Shellfish Immunol..

[75] Karaulov A.V., Renieri E.A., Smolyagin A.I., Mikhaylova I.V., Stadnikov A.A., Begun D.N., Tsarouhas K., Djordjevic A.B., Hartung T., Tsatsakis A. (2019). Long-term effects of chromium on morphological and immunological parameters of Wistar rats. Food Chem. Toxicol..

